# Using clinical guidelines to assess the potential value of laboratory medicine in clinical decision-making

**DOI:** 10.11613/BM.2021.010703

**Published:** 2020-12-15

**Authors:** Allan J. Hicks, Zoe L. Carwardine, Mike J. Hallworth, Eric S. Kilpatrick

**Affiliations:** 1 School of Medical Science, Griffith University, Southport, Australia; 2 Clinical Biochemistry, Sidra Medicine, Doha, Qatar; 3 Clinical Biochemistry, Royal Shrewsbury Hospital, Shrewsbury, UK; 4 Department of Clinical Biochemistry, Manchester Royal Infirmary, Manchester University NHS Foundation Trust, Manchester, UK

**Keywords:** cardiovascular diseases, guideline, clinical laboratory testing

## Abstract

**Introduction:**

It is often quoted that 70% of clinical decisions are based on laboratory results, but the evidence to substantiate this claim is lacking. Since clinical guidelines aim to document best-practice decision making for specific disease conditions, inclusion of any laboratory test means that the best available evidence is recommending clinicians use it. Cardiovascular disease (CVD) is the world’s most common cause of mortality, so this study reviewed all CVD guidelines published by five national/international authorities to determine what proportion of them recommended laboratory testing.

**Materials and methods:**

Five leading CVD guidelines were examined, namely the European Society of Cardiology (ESC), the UK National Institute for Health and Clinical Excellence (NICE), the American College of Cardiology (ACC), the Australian Heart Foundation (AHF) and the Cardiac Society of Australia and New Zealand (CSANZ).

**Results:**

A total of 101 guidelines were reviewed. Of the 33 individual ESC guidelines relating to CVD, 24/33 made a direct reference to the use of clinical laboratory tests in either diagnosis or follow-up treatment. The same applied to 15/20 of NICE guidelines, 24/32 from the ACC and 15/16 from the AHF/CSANZ. Renal function and blood count testing were the most recommended (39 and 26 times), with lipid, troponin and natriuretic peptide measurement advocated 25, 19 and 19 times respectively.

**Conclusions:**

This study has shown that laboratory testing is advocated by between 73% and 94% of individual CVD guideline recommendations from five national/international authorities. This provides an index to assess the potential value of laboratory medicine to healthcare.

## Introduction

The contribution of laboratory medicine to patient diagnosis, management and follow-up has proven difficult to quantify with systematic evidence of improved patient outcomes scarce (*1*). The phrase that ‘laboratory medicine influences 70% of clinical decisions’, or similar, has been published many times but the evidence to substantiate this claim is lacking. An editorial in the Annals of Clinical Biochemistry stated that the 70% figure was first published in 1996 and was based on anecdotal evidence and unpublished studies (*2*). The editorial lists various examples of the use of this phrase, albeit with slight modifications, for example; Lord Carter’s report on the UK Pathology service, the First Report of the UK House of Commons Select Committee on Health, and in the UK Department of Health report “Modernizing Pathology Services” (*2*).

A related and also oft-quoted statistic is that 70% of the electronic patient record is composed of laboratory data, but the main limitation of this observation is that the presence of laboratory results in a patient record does not necessarily equate to it being used in any clinical decision-making process (*3*). Over-requesting of testing is a common feature of many healthcare systems and panels of tests may include many analytes which are unrelated to the patient’s clinical condition. A 2016 study of laboratory use by oncologists and cardiologists found that 75% of all their patients underwent laboratory testing, and that this testing led to a substantial clinical decision in 66% of the patients (*4*).

Over time the 70% claim has apparently gained legitimacy simply due to the number of times that it had been repeated. Partly to examine this claim more closely, the International Federation of Clinical Chemistry and Laboratory Medicine (IFCC) established a Task Force on the Impact of Laboratory Medicine on Clinical Management and Outcomes in 2012. Its purpose was twofold: first, to evaluate the available evidence supporting the impact of laboratory medicine in healthcare; and secondly to develop a study design methodology for new retrospective and prospective studies capable of generating evidence to determine the contribution made by laboratory medicine to healthcare.

The IFCC Task Force published a summary of its findings in 2015 which included as one of its suggestions that the contents of authoritative clinical guidelines could provide an objective means of assessing the role of laboratory medicine in the management of specific health conditions (*1*). Clinical guidelines are documents which aim to guide decisions regarding diagnosis, management and treatment in specific areas of health care. By using the best evidence available the assumption is that each individual guideline recommendation is how clinicians should be making their clinical decisions.

In respect to which health condition should have its guidelines examined, according to the World Health Organization (WHO) cardiovascular disease (CVD) is the world’s most prevalent cause of mortality encompassing a large number of diseases including those of coronary heart disease, heart failure, rhythm and valvular abnormalities as well as cerebrovascular diseases (*5*). The report estimated that 17.9 million people worldwide die from CVDs which equates to 31% of all deaths and, of these, 85% were due to myocardial infarction or stroke (*5*). The British Heart Foundation report that CVD accounts for almost 170,000 deaths in the UK costing the National Health Service there £6.8 billion in 2012/2013 (*6*). In Europe, CVD is responsible for 3.9 million (45%) of all deaths annually (*7*). The American Heart Association has reported that coronary heart disease is the leading cause of death among Americans, accounting for 840,678 (30%) deaths in 2016 (*8*). According to the Australian Institute of Health and Welfare Alliance, CVD directly contributed to 45,400 deaths in 2015 which accounts for 29% of all deaths that year in Australia (*9*).

Accordingly, this study has chosen to examine some national and international cardiovascular clinical guidelines in detail from the countries just mentioned in order to determine what proportion of them recommended laboratory testing.

## Materials and methods

We conducted a review of all the individual cardiovascular guidelines available on the websites of the UK National Institute for Health and Care Excellence (NICE), the European Society of Cardiology (ESC), the American College of Cardiology (ACC), and Cardiac Society of Australia and New Zealand (CSANZ) and Australian Heart Foundation (AHF) in their online versions which were current on the 23rd April 2020 (*10*-*14*). All the guidelines from the cardiology societies were examined while the NICE guidelines were limited only to those categorised by them as referring to ‘cardiovascular conditions’. The AHF and the CSANZ guidelines were combined because a number of their guidelines were shared and, where they were identical, only one was included in the analysis statistics. These specific national/international guidelines were chosen because of their geographical spread and because they have previously been found to be rigorous with, for example, NICE and ESC guidance having the highest Appraisal of Guidelines for Research and Evaluation (AGREE) II scores for cardiovascular risk assessment in a systematic review (*15*).

Each individual guideline was read through in its entirety to determine if it was documented that laboratory involvement (defined as a test usually associated with one of the Laboratory Medicine disciplines, Histology or Genetics) was required for either an initial diagnosis or the ongoing management of care of the cardiovascular condition referred to in the document. Data was collected separately for tests which were used for initial diagnosis compared to those required for follow-up, and all forms of testing were included, not just analyses traditionally associated with CVD.

The proportion of guidelines containing laboratory medicine recommendations were determined for each national/international authority (AHF and CSANZ regarded as one) and for them combined. These proportions were calculated by simply dividing the number of individual guidelines indicating testing (if used for diagnosis and/or follow-up this was counted as one) by the total number of guidelines, whether for each national/international authority or for all authorities combined.

For solely the purpose of determining which types of tests were mentioned most frequently by the guidelines, some related tests were categorised together. Thus, renal function related testing included ‘renal function tests’, ‘creatinine’ and ‘eGFR’ in the text; natriuretic peptides included ‘natriuretic peptides’, ‘BNP’ and ‘NT-proBNP’; ‘calcium’, if mentioned separately from other electrolytes, was included in an "electrolytes group" and FBC and CBC were regarded as synonyms. Genetic and histology tests were not subcategorised any further.

As this is a review of existing guidelines, no Institutional Review Board approval was required.

## Results

Table 1 shows the 101 guidelines related to CVD available from NICE, ESC, ACC and the AHF/CSANZ and summarises the number and/or percentage indicating pathology testing for either initial diagnosis, follow up pathology testing or a combination of both. Taken together, *in vitro* diagnostic testing was required for diagnosing 64% of the conditions alluded to in the cardiovascular guidelines, with a similar proportion (62%) advocating testing for the continued management of such patients. As tests were sometimes recommended for both the diagnosis and follow-up of same condition, it means some form of testing was, on average, required by 77% of individual guidelines.

**Table 1:** Summary of requirement for *in vitro* diagnostic testing as part of individual cardiovascular guideline recommendations from 5 sources

**Organisation** **(Country/Region)**	Testing required for initial diagnosis*	**Testing required for further management**	**Guidelines requiring any testing**	**Total number of CVD guidelines**
NICE (UK)	10	13	15	20
ESC (Europe)	20	17	24	33
ACC (USA)	20	24	24	32
AHF/CSANZ (Australasia)	15	9	15	16
Total, N (%)	65 (64)	63 (62)	78 (77)	101 (100)
*All refer to the number of guidelines requiring testing. NICE - UK National Institute for Health and Clinical Excellence. ESC - European Society of Cardiology. ACC - American College of Cardiology. AHF - Australian Heart Foundation. CSANZ - Cardiac Society of Australia and New Zealand. CVD - cardiovascular disease.				

Tables 2-5 respectively show details of the individual NICE, ESC, ACC and the AHF/CSANZ guidelines including the year published and the tests mentioned within the body of the document. The test names used in these tables are as described in the respective guidelines.

**Table 2:** National Institute for Health and Clinical Excellence (NICE) Guidelines

**Guideline**	**Year published**	**Testing required for initial diagnosis**	**Testing required for further management**	**Indicated test(s)**
Hypertension in pregnancy: diagnosis and management	2019	Yes	Yes	RFT, LFT, FBC, urinalysis
Chronic heart failure in adults: management	2018	Yes	Yes	NT-proBNP, RFT, LFT, FBC, lipids, HbA1c, TFT, urinalysis
Stable angina: management	2016	Yes	Yes	cTn, FBC
Hypertension in adults: diagnosis and management	2019	No	Yes	RFT, eGFR, Lipids, HbA1c, creatinine, urinalysis
Hyperglycaemia in acute coronary syndromes: management	2016	No	Yes	Glucose, HbA1c
Venous thromboembolic diseases: diagnosis, management and thrombophilia testing	2020	Yes	Yes	D-dimer, PT, APTT, FBC, RFT, LFT
Peripheral arterial disease: diagnosis and management	2018	No	No	/
Stroke rehabilitation in adults	2013	No	No	/
Myocardial infarction with ST-segment elevation: acute management	2013	No	No	/
Varicose veins: diagnosis and management	2013	No	No	/
Atrial fibrillation	2014	No	Yes	LFT, INR, digoxin concentrations
Cardiovascular Disease: risk assessment and reduction, including lipid modification	2016	Yes	Yes	eGFR, lipids, albumin, HbA1c, LFT, RFT, CK
Acute Heart Failure: diagnosis and management	2014	Yes	Yes	BNP, RFT, NT-proBNP
Prophylaxis against infective endocarditis: antimicrobial prophylaxis against infective endocarditis in adults and children undergoing interventional procedures	2016	No	No	/
Stroke and transient ischaemic attack in over 16s: diagnosis and initial management	2019	No	Yes	INR, glucose, HbA1c,
Familial hypercholesterolemia: identification and management	2019	Yes	Yes	Lipids
Venous thromboembolism in over 16s: reducing the risk of hospital-acquired deep vein thrombosis or pulmonary embolism	2019	No	No	eGFR
Unstable angina and Non-ST-elevation myocardial infarction: early management	2013	No	Yes	cTn, creatinine, glucose, FBC
Recent-onset chest pain of suspected cardiac origin: assessment and diagnosis	2016	Yes	No	cTn
Myocardial Infarction: cardiac rehabilitation and prevention of further MI	2013	No	Yes	RFT
RFT - renal function tests. LFT - liver function tests. FBC - full blood count. NT-proBNP - N-terminal pro-B-type natriuretic peptide. HbA1c - haemoglobin A1c. TFT - thyroid function tests. cTn - cardiac troponins. eGFR – estimated glomerular filtration rate. PT - prothrombin time. APTT - activated partial thromboplastin time. INR - international normalized ratio. CK - creatine kinase. BNP – B-type natriuretic peptide.				

**Table 3:** European Society of Cardiology (ESC) guidelines

**Guidelines**	**Year published**	**Testing required for initial diagnosis**	**Testing required for further management**	**Indicated test(s)**
Infective Endocarditis (Guidelines on Prevention, Diagnosis and Treatment of)	2015	Yes	Yes	CRP, ESR, IS, BC, creatinine, bilirubin, CBC
Ventricular Arrhythmias and the Prevention of Sudden Cardiac Death	2015	No	Yes	Electrolytes,
Pericardial Diseases (Guidelines on the Diagnosis and Management of)	2015	Yes	Yes	CRP, CBC, ESR, CK, cTn, RFT, LFT
Acute Coronary Syndromes in patients presenting without persistent ST-segment elevation	2015	Yes	Yes	cTn, lipids
Pulmonary Hypertension (Guidelines on Diagnosis and Treatment of)	2015	Yes	Yes	RFT, CBC, iron studies, LFT
Hypertrophic Cardiomyopathy	2014	Yes	No	BNP, cTn, CK, TFT, RFT, CBC,
Aortic Diseases	2014	Yes	No	BNP, cTn
ESC/EACTS Guidelines in Myocardial Revascularisation (Guidelines for)	2018	No	No	/
Acute Pulmonary Embolism (Diagnosis and Management of)	2019	Yes	No	D-dimer
ESC/ESA Guidelines on non-cardiac surgery: cardiovascular assessment and management	2014	Yes	No	cTn
Diabetes, Pre-Diabetes and Cardiovascular Diseases developed with the EASD	2019	Yes	Yes	Glucose, lipids, HbA1c
Stable Coronary Artery Disease (Management of)	2019	Yes	Yes	Glucose, LFT, TFT, CBC, lipids, HbA1c, CK, creatinine
Cardiac Pacing and Cardiac Resynchronization Therapy	2013	No	No	
Arterial Hypertension (Management of)	2018	Yes	Yes	Glucose, LFT, RFT, eGFR, lipids, creatinine
Valvular Heart Disease (Management of)	2017	Yes	Yes	BNP
Atrial Fibrillation (Management of) 2010 and Focused Update (2012)	2016	No	No	/
Acute Myocardial Infarction in patients presenting with ST-segment elevation (Management of)	2017	Yes	No	cTn
Acute and Chronic Heart Failure	2016	Yes	Yes	BNP
CVD Prevention in clinical practice (European Guidelines on)	2016	No	No	/
Dyslipidaemias (Management of)	2019	Yes	Yes	Lipids, CRP
Cardiovascular Diseases during Pregnancy (Management of)	2018	Yes	Yes	CBC, RFT, LFT, BNP, cTn, D-dimer, urine protein
Peripheral Artery Diseases (Diagnosis and Treatment of)	2017	Yes	Yes	Glucose, lipids, creatinine, urine protein, CBC, RFT, HbA1c,
Grown-Up Congenital Heart Disease (Management of)	2010	No	Yes	CBC, ferritin, creatinine, uric acid, BNP, folic acid, vitamin B12
Device Therapy in Heart Failure (Focused Update)	2010	No	No	/
Syncope (Guidelines on Diagnosis and Management of)	2018	No	No	/
The Role of Endomyocardial Biopsy in the Management of Cardiovascular Disease	2007	Yes	No	Histological examination
B-Adrenergic Receptor Blockers (Expert Consensus Document on)	2004	No	No	/
Angiotensin Converting Enzyme Inhibitors in Cardiovascular Disease (Expert Consensus Document on)	2004	No	Yes	RFT, creatinine
Antiplatelet Agents (Expert Consensus Document on the Use of)	2004	No	No	/
Supraventricular Arrhythmias (ACC/AHA/ESC Guidelines for the Management of Patients with)	2003	No	Yes	TFT
Estimation of ten-year risk of fatal cardiovascular disease in Europe: the SCORE project	2003	Yes	No	Lipids
Neonatal Electrocardiogram (Guidelines for the interpretation of the)	2002	No	No	/
Chest Pain (Management of)	2002	Yes	Yes	cTn, CK
CRP - C-reactive protein. ESR - erythrocyte sedimentation rate. IS - infectious serology. BC - blood culture. CBC – complete blood count. CK - creatine kinase. cTn - cardiac troponins. RFT - renal function tests. LFT - liver function tests. BNP – B-type natriuretic peptide. TFT - thyroid function tests. HbA1c - haemoglobin A1c. eGFR – estimated glomerular filtration rate.				

**Table 4:** American College of Cardiology (ACC) Guidelines

**Guideline**	**Year published**	**Testing required for initial diagnosis**	**Testing required for further management**	**Indicated test(s)**
Focused Update of the 2014 AHA/ACC/HRS Guideline for the Management of Patients With Atrial Fibrillation	2019	No	Yes	CRP, CBC, TFT, RFT, LFT, electrolytes, INR, coagulation monitoring
Guideline on the Management of Blood Cholesterol	2018	Yes	Yes	Lipids
Guideline on the Evaluation and Management of Patients With Bradycardia and Cardiac Conduction Delay	2018	No	No	/
Guideline for the Management of Adults With Congenital Heart Disease	2018	No	Yes	APTT, INR, BNP
Guideline for Management of Patients With Ventricular Arrhythmias and the Prevention of Sudden Cardiac Death	2017	Yes	Yes	BNP, cTn, electrolytes, lipids, calcium
Prevention, Detection, Evaluation, and Management of High Blood Pressure in Adults	2017	Yes	Yes	Fasting glucose, CBC, lipids, creatinine, eGFR, Ca2+, electrolytes, TFT, urinalysis
Focused Update of the 2013 ACCF/AHA Guideline for the Management of Heart Failure	2017	Yes	Yes	BNP, cTn, eGFR, iron
Focused Update of the 2014 AHA/ACC Guideline for the Management of Patients With Valvular Heart Disease	2017	Yes	Yes	BNP, cTn, electrolytes, RFT, iron
Guideline for the Evaluation and Management of Patients With Syncope	2017	Yes	Yes	CBC, electrolytes, BNP, cTn, glucose
Guideline on the Management of Patients With Lower Extremity Peripheral Artery Disease	2016	No	Yes	Fasting glucose, RFT, HbA1c
Guideline Focused Update on Duration of Dual Antiplatelet Therapy in Patients With Coronary Artery Disease	2016	No	No	/
Focused Update on Primary Percutaneous Coronary Intervention for Patients With ST-Elevation Myocardial Infarction	2015	No	No	/
Surgery for Aortic Dilatation in Patients With Bicuspid Aortic Valves	2015	No	No	/
Guideline for the Management of Adult Patients With Supraventricular Tachycardia	2015	No	No	/
Guideline for the Management of Patients With Non–ST-Elevation Acute Coronary Syndromes	2014	Yes	Yes	cTn, BNP, lipids, RFT fasting glucose, HbA1c, creatinine, eGFR,
Strategies to Enhance Application of Clinical Practice Guidelines in Patients With Cardiovascular Disease and Comorbid Conditions	2014	No	No	/
Guideline on Perioperative Cardiovascular Evaluation and Management of Patients Undergoing Noncardiac Surgery	2014	No	Yes	Natriuretic peptides
Guideline for the Diagnosis and Management of Patients With Stable Ischemic Heart Disease	2012	Yes	Yes	Lipids, HbA1c, creatinine
Guideline for the Management of Patients with Atrial Fibrillation	2014	Yes	Yes	CRP, CBC, TFT, RFT, LFT, electrolytes, INR, coagulation monitoring
Guideline for the Management of Patients with Valvular Heart Disease	2014	No	Yes	eGFR, BNP, INR, LFT, BC, IS, histology, RF
Peripheral Arterial Disease (Lower Extremity, Renal, Mesenteric, and Abdominal Aortic)	2013	No	Yes	Fasting glucose, RFT, HbA1c
Guideline on the Assessment of Cardiovascular Risk	2013	Yes	Yes	CRP, creatinine, eGFR
Guideline for the Management of Heart Failure	2013	Yes	Yes	Fasting glucose, CBC, lipids, creatinine, eGFR, Ca2+, electrolytes, TFT, urinalysis
ST-Elevation Myocardial Infarction	2012	Yes	Yes	Lipids, glucose, HbA1c, cTn, RFT, coagulation monitoring
Guideline for the Diagnosis and Treatment of Hypertrophic Cardiomyopathy	2011	Yes	Yes	cTn, genetic testing
Guideline for Coronary Artery Bypass Graft Surgery	2011	No	No	/
Guideline for Percutaneous Coronary Intervention	2011	Yes	Yes	Glucose, HbA1c, RFT, cTn, BNP, iron, lipids
Secondary Prevention and Risk Reduction Therapy for Patients With Coronary and Other Atherosclerotic Vascular Disease	2011	Yes	Yes	lipids, RFT, electrolytes,
Cardiovascular Disease Prevention in Women	2011	Yes	Yes	Lipids, glucose, HbA1c, hormone concentrations
Extracranial Carotid and Vertebral Artery Disease	2011	Yes	Yes	CBC, Ca2+, lipids, glucose, HbA1c
Thoracic Aortic Disease	2010	Yes	Yes	Genetic testing, IS, BC, D-dimer, CRP, CBC, coagulation monitoring, blood type and screen, urinalysis
Device-Based Therapy of Cardiac Rhythm Abnormalities	2008	No	No	/
CRP - C-reactive protein. CBC – complete blood count. TFT - thyroid function tests. RFT - renal function tests. LFT - liver function tests. INR - international normalized ratio. APTT - activated partial thromboplastin time. BNP – B-type natriuretic peptide. cTn - cardiac troponins. eGFR – estimated glomerular filtration rate. Ca2+ - ionized calcium. HbA1c - haemoglobin A1c. BC - blood culture. IS - infectious serology. RF - rheumatoid factor. IS - infectious serology.				

**Table 5:** Australian Heart Foundation (AHF) and the Cardiac Society of Australia and New Zealand (CSANZ) Guidelines

**Guideline**	**Source**	**Year Published**	**Testing required for initial diagnosis**	**Testing required for further management**	**Indicated test(s)**
Australian Clinical Guidelines for the Diagnosis and Management of Atrial Fibrillation	AHF/CSANZ	2018	Yes	Yes	CBC, RFT, TFT, HbA1c, INR, electrolytes, lipids
Guidelines for the Prevention, Detection, and Management of Heart Failure in Australia	AHF/CSANZ	2018	Yes	Yes	BNP, genetic testing, RFT, creatinine, glucose, CBC
Coronary Artery Calcium Scoring	CSANZ	2017	Yes	No	eGFR, glucose, HbA1c, lipids,
Clinical Guideline for the diagnosis and management of hypertension in adults	AHF	2016	Yes	Yes	Urinalysis, RFT, glucose, eGFR, creatinine, lipids, CBC
Guidelines for the Diagnosis and Management of Catecholaminergic Polymorphic Ventricular Tachycardia (CPVT)	CSANZ	2016	No	Yes	Genetic testing
Clinical guidelines for the management of Acute coronary syndrome	AHF/CSANZ	2016	Yes	Yes	cTn, lipids, glucose, HbA1c, eGFR, APTT, CBC
Diagnosis and Management of Familial Dilated Cardiomyopathy	CSANZ	2016	Yes	No	CK, genetic testing
Diagnosis and Management of Familial Hypercholesterolaemia	CSANZ	2016	Yes	Yes	Lipids, genetic testing
Update on the Diagnosis and Management of Familial Long QT Syndrome	CSANZ	2016	Yes	No	Genetic testing
Diagnosis and Management of Hypertrophic Cardiomyopathy	CSANZ	2016	Yes	No	Histological examination, genetic testing
Update on the diagnosis and management of inherited aortopathies, including Marfan syndrome	CSANZ	2016	Yes	No	genetic testing
The routine cardiac assessment of newborns with Down syndrome	CSANZ	2016	No	No	/
Position Statement on the Diagnosis and Management of Brugada Syndrome	CSANZ	2015	Yes	No	Genetic testing
Guidelines for the management of Absolute cardiovascular disease risk	AHF/CSANZ	2012	Yes	Yes	Urinalysis, RFT, glucose, eGFR, creatinine, lipids, CBC
Guideline for prevention, diagnosis and management of acute rheumatic fever and rheumatic heart disease	AHF/CSANZ	2012	Yes	Yes	CBC, CRP, BC, IS, ESR
Guidelines for the diagnosis and management of Arrhythmogenic Right Ventricular Cardiomyopathy	CSANZ	2019	Yes	No	Genetic testing
CBC – complete blood count. RFT - renal function tests. TFT - thyroid function tests. HbA1c - haemoglobin A1c. INR - international normalized ratio. BNP – B-type natriuretic peptide. eGFR – estimated glomerular filtration rate. cTn - cardiac troponins. APTT - activated partial thromboplastin time. CK - creatine kinase. CRP - C-reactive protein. BC - blood culture. IS - infectious serology. ESR - erythrocyte sedimentation rate.					

Twenty seven different test categories were explicitly mentioned 276 times within the 78 guidelines requiring testing (Tables 2-5). The commonest were renal function related biochemistry (mentioned 39 times), complete (or ‘full’) blood counts (26 times), lipids (25 times) and blood/plasma glucose (20 times). More specific to cardiovascular disease were the troponins (19 times) and natriuretic peptides (19 times).

## Discussion

Guidelines are designed to inform optimum decision-making by clinicians, and therefore provide a measure of the value of laboratory medicine. This study has shown that between 73% and 94% of individual UK (NICE), European (ESC), United States (ACC) and Australasian (AHF/CSANZ) guidelines related to CVD state that laboratory testing of some sort is recommended. The topics of these individual guidelines tend to be related to specific clinical conditions, so it means that, taken together, 77% of clinical recommendation pathways involving each of these cardiovascular diseases require laboratory assistance at some point in their diagnosis or management.

There are both limitations and strengths to using guidelines as a measure of laboratory medicine’s contribution to healthcare. One potential limitation is that clinical guidelines are not necessarily followed by all physicians or other healthcare staff. A clinician’s own pathway for patients may therefore involve more or less testing than is being recommended. However, the main advantage of our method is that at least the guidelines define an evidence-based recommendation for best practice in each specific clinical scenario. The guidelines also tend to be specific in the test or tests that are recommended, which is advantageous in two ways. First, it helps ensure that the contribution of laboratory medicine to healthcare is not exaggerated by over-requesting - as could be the case if health records were solely being examined - and, secondly, that a clinical decision relevant to the health condition is intended to be taken on the basis of the result.

It should be noted that the guidelines examined in this study were produced in relatively affluent countries with developed healthcare systems and so the use of laboratory medicine testing advocated in other, less wealthy, countries may well differ. Nonetheless, this does not preclude the same methodology as used here being applied to any alternative CVD guidelines from other countries.

Beyond CVD, there is also the possibility that the same objective approach to guidelines could be applied to determine the contribution of laboratory medicine in other, less prevalent, disease groups. Combining the findings from these different disease groups could potentially provide a more accurate overall assessment of the significance of laboratory medicine to healthcare in general.

In summary, this study has found a requirement for the use of laboratory testing in 73% to 94% of cardiovascular guidelines produced by five different organisations. It does not provide a direct link to improved patient outcomes but does provide an index to assess the potential value of laboratory medicine to healthcare which can complement other metrics.
